# Performance Analysis of Uplink Code Division Multiplexing for LEO Satellite Constellations Under Nonlinear Power Amplifiers

**DOI:** 10.3390/s24216879

**Published:** 2024-10-26

**Authors:** Monica Visintin, Riccardo Schiavone, Roberto Garello

**Affiliations:** Department of Electronics and Telecommunications (DET), Politecnico di Torino, 10129 Torino, Italy; monica.visintin@polito.it (M.V.); riccardo.schiavone@polito.it (R.S.)

**Keywords:** LEO satellites, ground station, telecommand, CDMA, high-power amplifier

## Abstract

This paper studies the performance of the communication link between a ground station and the satellites of a LEO constellation, employing code division multiplexing and a non-linear high-power amplifier. The analysis shows that the input power selection at the high-power amplifier of the ground station has a significant impact on overall system performance. The results concerning output power, the challenge of adjusting the back-off with a continuously changing number of satellites, and improved energy efficiency suggest operating in saturation. In this scenario, we can choose to transmit directly the sign of the sum of the signals directed to individual satellites. Analytical exact and simplified results are derived, enabling the estimation of performance as a function of the number of satellites being served when the amplifier operates at saturation. These analytic results are further validated through simulations. A formula to compute the loss across different numbers of satellites is also presented. The performance under saturated amplifier conditions is evaluated, compared, and discussed, providing valuable insights for simplifying the design and operation of satellite uplink communication systems under power amplifier constraints.

## 1. Introduction

Low Earth orbit (LEO) satellite constellations are becoming increasingly important for a wide range of applications. Non-terrestrial networks (NTNs) will integrate satellite communication into the 6G architecture, providing global connectivity [1,2,3,4]. Mega-constellations such as Starlink, OneWeb, and IRIS^2^ are playing (or will play) a crucial role in providing broadband internet to users on Earth, helping to bridge the digital divide by bringing connectivity to remote and underserved areas [5]. Their use has also been proposed for serving LEO satellites with a multi-layer spac network approach [6,7,8,9]. In addition to broadband services, LEO constellations are also important for enabling the Internet of Things (IoT) by providing global coverage for connected devices [10,11,12,13,14]. Moreover, they are extensively used in remote sensing and Earth observation, offering valuable data for environmental monitoring, disaster management, and resource tracking [15,16]. In the coming decades, we can expect the launch of several proprietary constellations, each comprising dozens or even hundreds of LEO small satellites [17].

For LEO constellations, Telecommand uplink operations managed by ground stations are crucial for the effective operation and maintenance of the satellite network [18,19]. The ability to remotely control and monitor the satellites from Earth is essential for ensuring the constellation’s performance and stability. Ground stations must constantly communicate with the satellites to manage their orbits, monitor their health, make real-time adjustments, avoid collisions, update sensor operations, and correct any drift. Therefore, effective ground-based control systems are a critical component in the successful deployment and operation of LEO satellite constellations.

Code division multiplexing (CDM) and code division multiple access (CDMA) [20] are among the most widely used techniques for LEO satellite constellation communication links [21,22,23] (In our papers, we typically differentiate between CDM, used for the uplink from the Ground Station to multiple satellites, and CDMA, used for the downlink from multiple satellites to the ground station.). Their popularity stems from the ability to serve multiple satellites simultaneously within the same frequency band by utilizing spreading sequences with good cross-correlation properties. These properties help to minimize interference among signals directed to different satellites, ensuring reliable communication and control. Furthermore, CDM and CDMA provide inherent protection against jamming, as the use of spread spectrum techniques makes it more difficult for unauthorized entities to disrupt the signal [24].

### Motivation and Contribution

In uplink communications, ground stations employ power amplifiers to boost the transmitted power levels, ensuring that the signal can reach multiple satellites in an LEO constellation. However, a significant challenge arises due to the non-linear nature of these amplifiers, which introduce distortion into the transmitted signal. This distortion can degrade the overall system performance, especially when multiple satellites are being served simultaneously using CDM. This could suggest operating in linearity by applying a back-off and introducing a loss in transmitted power. Nevertheless, a key characteristic of LEO constellations is that the number of visible satellites continuously changes, as the satellites move rapidly and remain visible for only short intervals. As a result, it is not feasible to design the ground station to serve a constant number of satellites at all times. This fact, combined with the tendency of a CDM signal to approximate a Gaussian distribution in amplitude when multiple signals are summed together, makes it extremely challenging to operate in linearity with a fixed back-off. The challenge is related to the peak-to-average power ratio (PAPR) of the CDM signal, which depends on the number of satellites. As the number of LEO satellites changes very rapidly, so does the PAPR, making it difficult to select an optimal back-off that ensures linearity across all conditions. Moreover, operating with a fixed back-off reduces the efficiency of the amplifier compared to when it operates in saturation. As a result, many ground stations operate directly in saturation. While the performance of satellite communications with an amplifier operating in saturation has been well investigated for the downlink, it has been less studied for the uplink, particularly when using CDMA.

In this paper, we build on the concept of transmitting the sign of the sum of the signals when the amplifier operates in saturation, a technique first introduced in [25]. The performance of the CDMA satellite uplink was analyzed through accurate simulations in [26]. The objective of this paper is to analytically study the performance of a CDM-based uplink to multiple satellites in the presence of a non-linear power amplifier operating in saturation. The main contributions of this paper are as follows:

- The study of the impact of input power level on system performance across different numbers of satellites.
- A first theorem for the exact calculation of error probability when the amplifier operates in saturation as a function of the number of satellites, including a simplified formula.
- The validation of exact and simplified analytical results through simulations.
- A second theorem for the analytical computation of loss based on the number of satellites served.
- A comprehensive analysis of the impact of amplifier non-linearities on system performance, along with guidelines for simplifying satellite uplink operation under power amplifier constraints.

This paper is organized as follows: In Section 2, we present the problem. In Section 3, we discuss the impact of the power amplifier in terms of the output power and bit error rate across different numbers of satellites. In Section 4, we present the theorem for analytically computing the system performance of a CDM system operating with a saturated amplifier, as a function of the number of satellites. Both exact and simplified expressions are presented. The analytic results are validated through simulation and discussed. In Section 5, we present a theorem for analytically computing the loss for a given number of satellites. These results are discussed, and practical considerations for the operation of the ground station uplink are provided. Section 6 concludes the paper, while the proofs of the theorems are provided in Appendixes Appendix A and Appendix B.

## 2. Problem Statement

**Figure 1:**
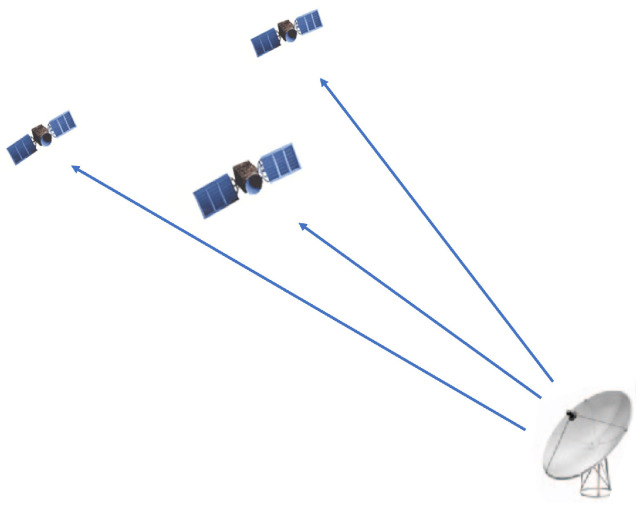
A ground station serving multiple satellites.

The scenario considered in this paper is illustrated in Figure 1. A ground station serves $N_{S}$ satellites simultaneously, on the same frequency band, using a CDM system. (The satellites may be in the same or different orbits and are at varying distances; the analysis is based on the $E_{b}/N_{0}$ ratio of each individual satellite).

### 2.1. CDM-Transmitted Signal

To each *i*-th satellite, we assign a spreading code of *L* chips

$${\underline{c}}_{i}^{\prime }={\left\{c_{i}^{\prime }\left(m\right)\in \left\{0,1\right\}\right\}}_{m=1}^{L},$$

which is mapped into a bipolar spreading sequence of *L* symbols

$${\underline{c}}_{i}={\left\{c_{i}\left(m\right)\in \left\{-1,1\right\}\right\}}_{m=1}^{L}$$

where $c_{i}\left(m\right)=2c_{i}^{\prime }\left(m\right)-1$. In general, we assume that the sequence $c_{i}\left(m\right)$ is periodic with period *L* longer than the spreading factor *M* (the number of chips per information bit). Moreover, we assume that $c_{i}\left(m\right)=1$ with a probability of 1/2, i.e., we model the spreading codes as pseudo-random sequences.

We focus on a base-band 2-PAM constellation with a rectangular waveform. The transmitted signal is equal to the following:

(1) $$s_{T}\left(t\right)=\sum_{i=0}^{N_{S}-1}\sum_{n=-\infty }^{\infty }\alpha_{i}b_{i}\left(n\right)P_{T_{b}}\left(t-nT_{b}\right)\sum_{m=-\infty }^{\infty }c_{i}\left(m\right)P_{T_{c}}\left(t-mT_{c}\right)$$

where we have the following:

- $N_{S}$ is the number of satellites currently served.
- $\alpha_{i}$ is the signal level that determines the power transmitted to user *i*, equal to $P_{i}=\alpha_{i}^{2}$. Hereafter, we will assume $\alpha_{i}=\alpha$, which is equal for all the users so that the average power of $s_{T}\left(t\right)$ is $P_{in}=N_{S}\alpha^{2}$.
- $b_{i}\left(n\right)$ is the *n*-th bipolar (+1/−1) symbol carrying information.
- $P_{T}\left(t\right)$ is the rectangular pulse with the unit amplitude for 0≤t<T and zero elsewhere.
- $T_{b}$ is the bit/symbol time.
- $c_{i}\left(m\right)$ is the *m*-th bipolar symbol corresponding to the binary spreading sequence chip $c_{i}^{\prime }\left(m\right)$ assigned to user *i*.
- $T_{c}$ is the chip time, where $T_{c}=T_{b}/M$, and *M* is the spreading factor.

**Figure 2:**
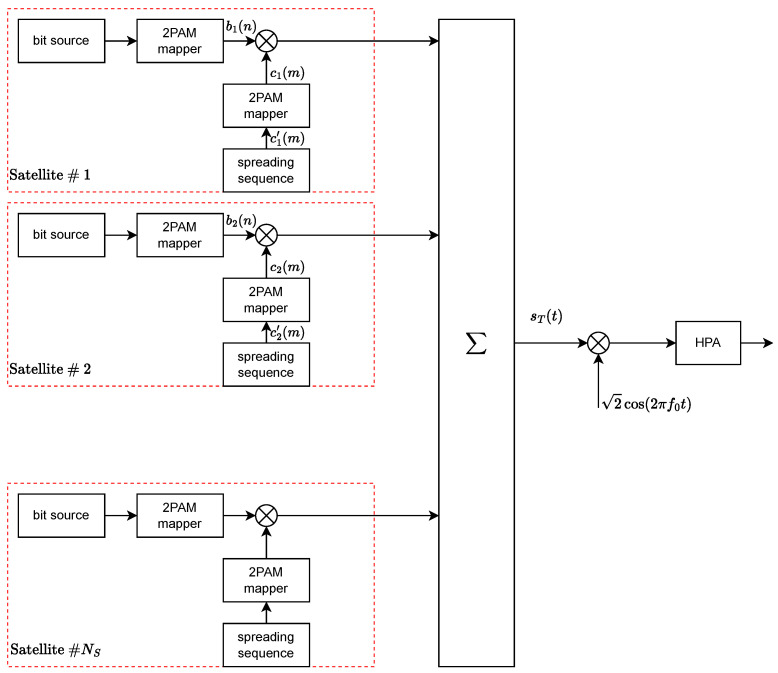
Transmitter block diagram.

The corresponding block diagram is shown in Figure 2 and $s_{T}\left(t\right)$ is the input of the frequency up-converter, which is followed by the high-power amplifier.

### 2.2. Power Amplifier

**Figure 3:**
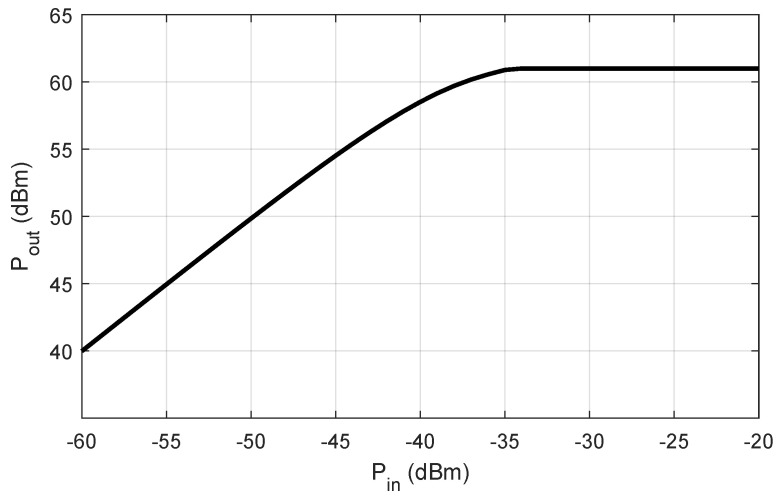
AM/AM curve for the considered high-power amplifier.

The AM/AM curve of the high-power amplifier (HPA) considered in this paper was derived from the specs available for the 2.5 kW SuperLinear Modular TWTA X-Band [27], which is characterized by these parameters:

- Gain equal to 100 dB, maximum output power 31 dBW, intermodulation with respect to the sum of 2 equal carriers 5 MHz apart −23.5 dBc max. at 400 W total output power.
- The input–output model is as follows:
  $$y\left(t\right)=\sqrt{G}x\left(t\right)+a_{3}x^{3}\left(t\right)+a_{5}x^{5}\left(t\right)$$
  where x(t) is the input RF signal, $G=10^{10}$ (corresponding to 100 dB).
- Coefficients $a_{3}$ and $a_{5}$ were found by measuring the intermodulation at several input powers and numerically finding the solution that gives intermodulation equal to −23 dBc and 400 W of output power. The fifth harmonic was necessary to obtain the maximum output power $P_{max}$ equal to 31 dBW (or 61 dBm).
- The HPA datasheet [27] specifies the following for the AM/PM conversion: “6.0°/dB max; with optional linearizer, can be tuned to 2.0°/dB max”. Given this limited information, we assumed a constant AM-PM curve, which might be reasonable in the presence of a linearizer. Further comments on this hypothesis and its impact on performance in terms of linearity and saturation will be discussed in the following:

The derived AM/AM curve is depicted in Figure 3.

## 3. Impact of Power Amplifier Nonlinearities on the System Performance

**Figure 4:**
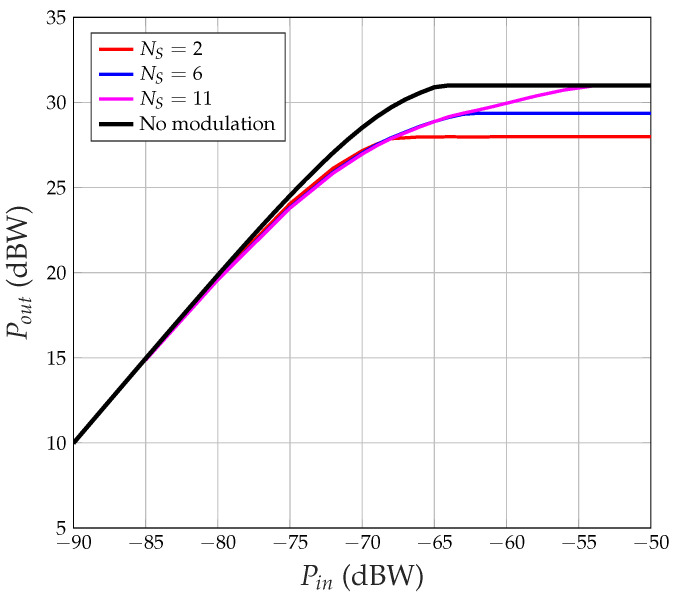
HPA output power versus input power for various numbers of satellites.

Our first goal is to study the impact of input power on system performance when HPA non-linearity is taken into consideration. The signal $s_{T}\left(t\right)$ from Equation (1) is used to drive the HPA with different nominal input powers $P_{in}$, and the corresponding output power $P_{out}$ is evaluated. The results are presented in Figure 4.

Observing Figure 4, we note the following:

- When $N_{S}=2$, the maximum output power is limited to 28 dBW (instead of $P_{max}=31$ dBW), and this maximum is reached when $P_{in}=-68$ dBW;
- When $N_{S}=6$, max $P_{out}=29.36$ dBW at $P_{in}=-62$ dBW;
- When $N_{S}=11$, and max $P_{out}=P_{max}=31$ dBW at $P_{in}=-54$ dBW.

There is clearly a gap between the output power when the input is a CDM signal and the output power when the input is a pure sinusoid (‘No modulation’ curve in Figure 4). The reason is that, when the input power is such that the HPA works completely at saturation, the input signal is clipped, i.e., it is as if the input signal were as follows:

(2) $$s_{T}^{\prime }\left(t\right)=\sqrt{\frac{P_{in}}{N_{S}}}\mathrm{sign}\left[\sum_{i=0}^{N_{S}-1}\sum_{n}b_{i}\left(n\right)P_{T_{b}}\left(t-nT_{b}\right)\sum_{m}c_{i}\left(m\right)P_{T_{c}}\left(t-mT_{c}\right)\right]$$

where sign(x) is the sign (or signum) function, equal to 1 if x>0, to −1 if x<0, to 0 if x=0. We can observe the following:

- When there are $N_{S}=2$ satellites, $s_{T}\left(t\right)$ is either equal to 0 (with a probability of 0.5) or to $\sqrt{\frac{P_{in}}{2}}$ (with a probability of 0.25) or to $-\sqrt{\frac{P_{in}}{2}}$ (with a probability of 0.25); therefore, the signal at the HPA output has either instantaneous power $P_{max}$ (with a probability of 0.5) or 0 (with a probability of 0.5); the average power at the HPA output is $P_{out,av}=P_{max}/2$ (31−3 = 28 dBW, as in Figure 4).
- When there are $N_{S}$ satellites, $s_{T}\left(t\right)$ is equal to zero with a probability of
  π˜0=NSNS/212NS
  if $N_{S}$ is even (at a given instant *t* half of the chips are +1, half are −1), whereas, ${\tilde{\pi }}_{0}=0$ when $N_{s}$ is odd. If the input power $P_{in}$ is large enough, the output instantaneous power is $P_{max}$ with a probability of ${\tilde{\pi }}_{1}=1-{\tilde{\pi }}_{0}$, and it is zero with a probability of ${\tilde{\pi }}_{0}$. The average output power is, therefore,
  $$P_{out,av}=\left(1-{\tilde{\pi }}_{0}\right)P_{max}$$
  and the ratio between $P_{max}$ and $P_{out,av}$ is
  $$10\log_{10}\frac{P_{max}}{P_{out,av}}=-10\log_{10}\left(1-{\tilde{\pi }}_{0}\right).$$
  This loss cannot be recovered, it is, at most, 3 dB when $N_{S}=2$, and it decreases when $N_{S}$ increases.
- When there are 6 satellites, x(t) is zero with a probability of
  π˜0=63126=6×5×43×2164=2064=0.3125.
  Assuming that, when x(t)≠0 (probability 0.6875), the HPA output is $\pm \sqrt{P_{max}}$, then the average output power is $P_{out,av}=0.6875\;P_{max}$ (31 − 1.627=29.37 dBW, against the measured 29.36 dBW in Figure 4).
- When there are 11 satellites, the probability that x(t)=0 is zero (there are 11 chips that add together, so the result can never be 0). The output average power $P_{out,av}$ can reach the maximum value $P_{max}$ provided that $P_{in}$ is large enough. In general, an odd number of satellites allows us to obtain the following: $P_{out,av}=P_{max}$.

**Figure 5:**
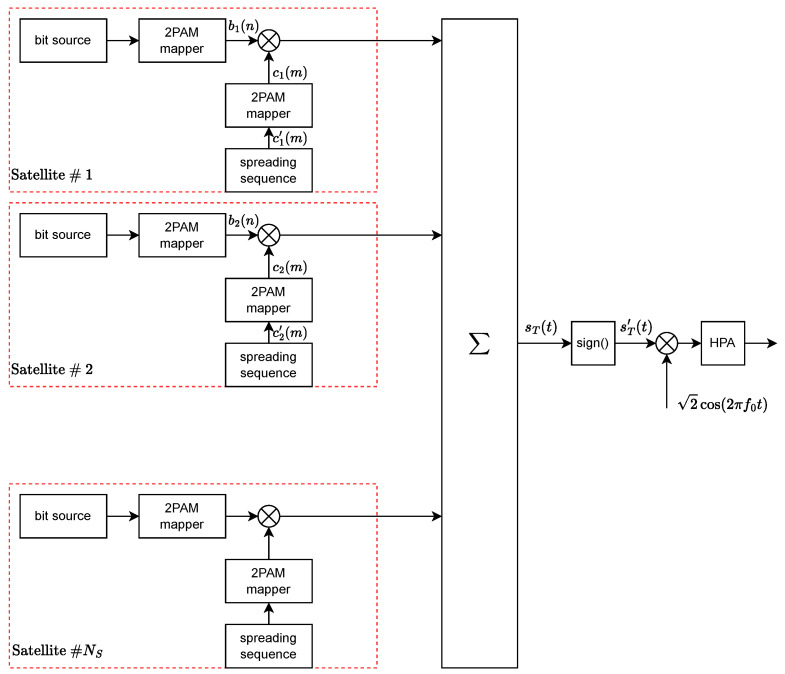
Equivalent scheme with the sign function for HPA working at saturation.

However, when $P_{out,av}=P_{max}$, it is as if we were transmitting the sign of the sum of the chips, instead of the sum of the chips. Consequently, in the following, we assume that the sign of the sum of the transmitted signals is sent, as shown in Figure 5. This approach was first proposed in [25]. As discussed later, this also provides advantages regarding potential distortions introduced by the AM/PM characteristic of the amplifier.

### BER Performance

**Figure 6:**
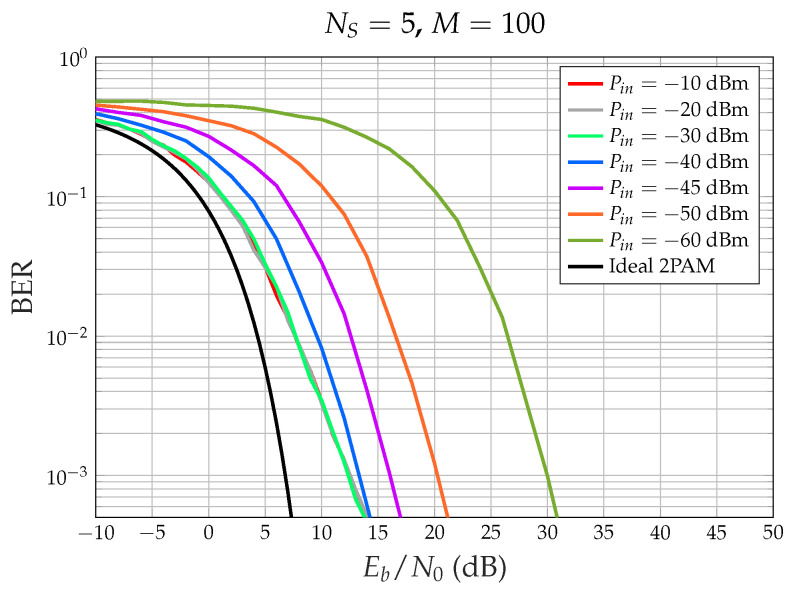
Simulated BER performance for $N_{S}=5$, spreading factor M=100, and different input powers $P_{in}$ of the HPA. Best performance for $P_{in}$= −10 dBm (saturation).

**Figure 7:**
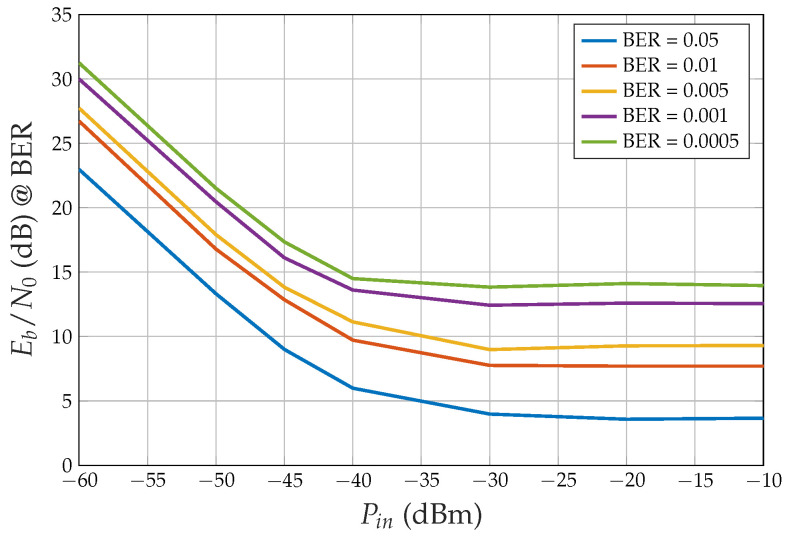
Simulated $E_{b}/N_{0}$ (dB) necessary to obtain the specified BER value versus the HPA input power $P_{in}$, $N_{S}=5$, spreading factor M=100. Best performance for $P_{in}$= −10 dBm (saturation).

Figure 6 shows the bit error rate (BER) performance for $N_{S}=5$ and several HPA input powers $P_{in}$. Note that we define $E_{b}=P_{max}T_{b}$, where $P_{max}=31$ dBW is the maximum power at the HPA output: $E_{b}$ changes if we change the HPA power, whereas $N_{0}$, which depends on the receiver, does not. We observe the following:

- When the HPA average input power is very small (−60 dBm), the amplifier works in its linear region, the output power is small and a much smaller $N_{0}$ is necessary to obtain a desired BER.
- On the other hand, when the HPA input power is large ([−20, −10] dBm), the amplifier works in the saturation region, and the output power is large, but interference arises among the CDM signal components, due to the hard limiting effect.
- When standard CDM without sign operation is transmitted, if the HPA introduces phase distortion (due to non-ideal AM/PM characteristics, which were not considered in the simulations), then the BER for the HPA linear region is higher than shown in Figure 6. On the contrary, when the sign is transmitted, the BER for $P_{in}$ larger than −30 dBm does not change since the output power is consistently equal to $P_{max}$ and the HPA-induced phase shift is also constant, as assumed in the simulations. Note that the receiver’s carrier phase recovery subsystem compensates for constant phase shifts.
- For desired BER values larger than $5\times 10^{-4}$, the minimum $E_{b}/N_{0}$ is obtained by driving the HPA at saturation (see Figure 7).

In an LEO satellite constellation, the number of satellites being served continuously changes because the satellites move rapidly, and the window of visibility is very short. The results suggest that instead of trying to operate in linearity by constantly adjusting the back-off, which depends on the number of satellites, and considering that with CDM the transmitted signal still maintains a Gaussian distribution of amplitudes, it is more efficient to operate directly in saturation. For this reason, having an analytical result that enables the calculation of BER performance for CDM with a saturated amplifier as a function of the number of satellites would be extremely useful. This is precisely the result presented in the next section.

**Figure 8:**
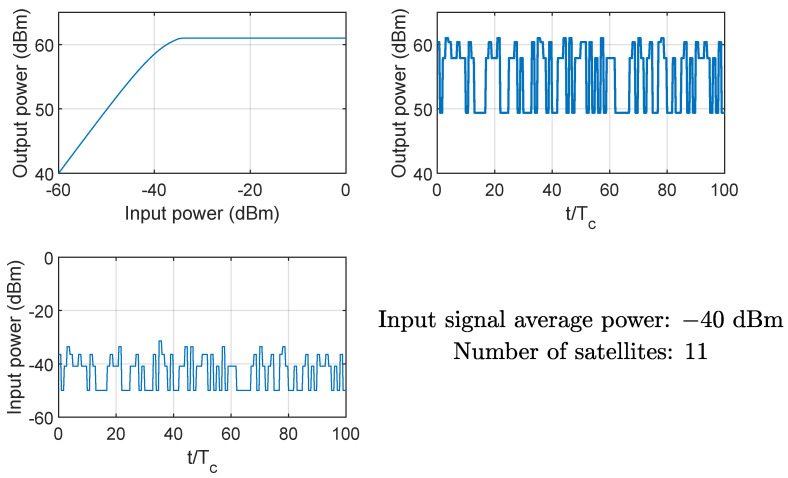
Example of HPA nonlinearity effects on the instantaneous power of the input and output signals; case of average input power equal to −40 dBm (linear region), 11 satellites.

**Figure 9:**
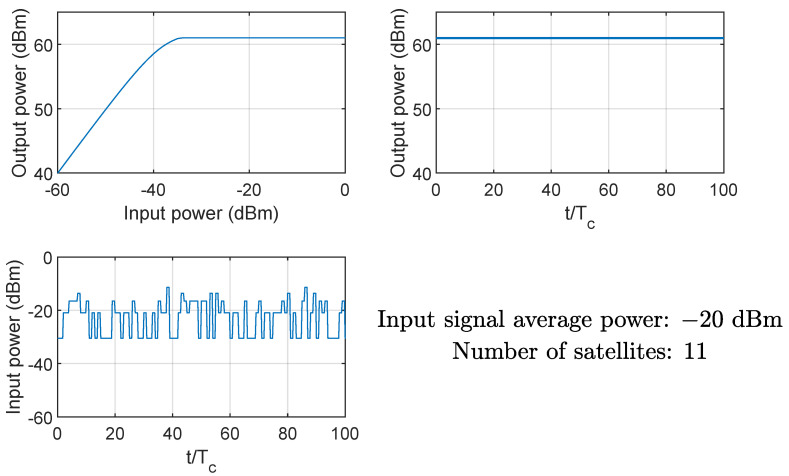
Example of HPA nonlinearity effects on the instantaneous power of the input and output signals; case of average input power equal to −20 dBm (nonlinear region), 11 satellites.

**Figure 10:**
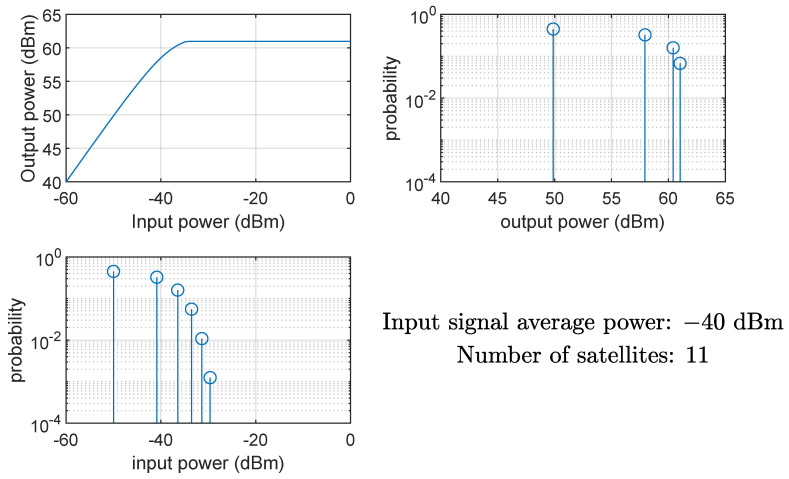
HPA nonlinearity effects on the probability mass function of the instantaneous power of the input and output signals; case of average input power equal to −40 dBm (linear region), 11 satellites.

**Figure 11:**
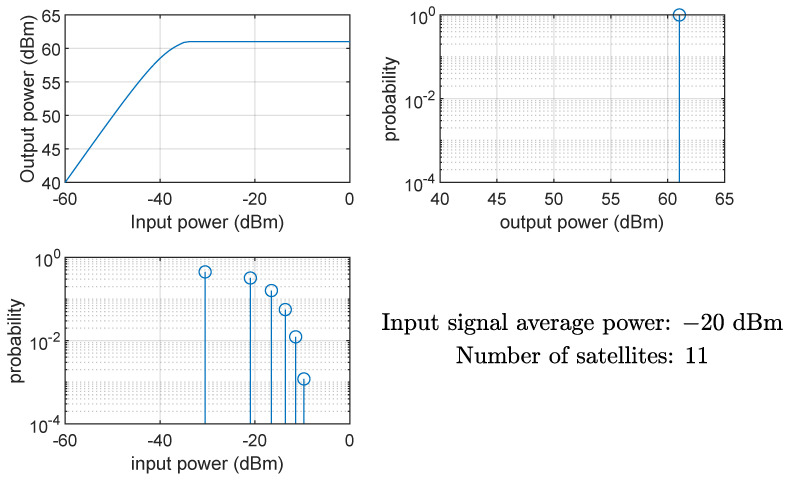
HPA nonlinearity effects on the probability mass function of the instantaneous power of the input and output signals; case of average input power equal to −20 dBm (nonlinear region), 11 satellites.

Figure 8 and Figure 9 show the effects on the instantaneous power of the signals at the input and output of the HPA when the average input power $P_{in}$ is equal to −40 dBm (linear region of the AM/AM curve) and −20 dBm (nonlinear region of the AM/AM curve), respectively. Clearly, if the HPA works in its nonlinear region, the output signal is compressed, and the instantaneous power is $P_{max}$. The same conclusion can be drawn from Figure 10 and Figure 11, showing the probability mass function of the input and output instantaneous power, for the HPA working in the linear and nonlinear regions, respectively.

## 4. The Analytical Performance of Uplink CDM-Serving Satellites with a Saturated Amplifier

As explained before, since it is convenient to drive the HPA at saturation, and since—in this condition—the signal is, in practice, hard-limited, this is equivalent to introducing a hard limiter (a sign function, as in Equation (2)) before the amplifier (see Figure 5). Such a transmitter was first proposed in [25]; however, to the best of our knowledge, no analytical result was provided for the CDM performance calculation with a non-linear amplifier working at saturation.

The baseband input of the HPA is as follows:

$$s_{T}^{\prime }\left(t\right)=\sqrt{P_{in}}\mathrm{sign}\left[\sum_{i=0}^{N_{S}-1}\sum_{n}b_{i}\left(n\right)P_{T_{b}}\left(t-nT_{b}\right)\sum_{m}c_{i}\left(m\right)P_{T_{c}}\left(t-mT_{c}\right)\right]$$

and the transmitted signal is (a complex envelope) as follows:

$$x\left(t\right)=\sqrt{2P_{out}}\mathrm{sign}\left[\sum_{i=0}^{N_{S}-1}\sum_{n}b_{i}\left(n\right)P_{T_{b}}\left(t-nT_{b}\right)\sum_{m}c_{i}\left(m\right)P_{T_{c}}\left(t-mT_{c}\right)\right]$$

where $P_{out}$ is the transmitted signal power when the HPA input power is $P_{in}$. The maximum value of $P_{out}$ is $P_{max}$ for a sufficiently large $P_{in}$ and an odd number of served satellites.

Note that, having introduced the hard limiter, the $E_{b}/N_{0}$ for a desired BER, in general, will be larger for the system in Figure 5 than for that in Figure 2. However, when $P_{in}$ is sufficiently large (i.e., HPA working in saturation, which as shown in the previous section is the best case), the two systems exhibit the same performance. The system depicted in Figure 5 has the advantage of allowing for a theoretical analysis, which is provided in the following theorem.

**Theorem** **1.**  *The error probability for an uplink CDM system serving $N_{S}$ satellites with random spreading codes and a saturated amplifier is as follows:*

- *For $N_{S}$ even, the exact error probability is as follows:*
(3) $$\begin{array}{cc} P_{b}\left(e\right)=\sum_{k_{0}=0}^{M}\sum_{k_{1}=0}^{M-k_{0}} & \frac{1}{2}erfc\left(\sqrt{\frac{N_{s}E_{b}}{M^{2}N_{0}}}\left(M-k_{0}-2k_{1}\right)\right)\\ & \times \frac{M!}{k_{0}!k_{1}!\left(M-k_{0}-k_{1}\right)!}\;\pi_{0}^{k_{0}}\pi_{m}^{k_{1}}\pi_{p}^{M-k_{0}-k_{1}}\;N_{S}\geq 4 \end{array}$$
(4) Pb(e)=∑k=0M12erfckNsEbM2N0Mk12MNS=2
  *and can be approximated by the following:*
(5) $$P_{b}\left(e\right)≃\frac{1}{2}erfc\sqrt{\frac{p_{0}^{2}N_{s}\left(E_{b}/N_{0}\right)}{1+2\left(1-p_{0}-p_{0}^{2}\right)\;N_{s}\left(E_{c}/N_{0}\right)}}$$
  *where*
Ec=Eb/M,p0=NS−1NS/2−12−(NS−1)
$$\pi_{0}=p_{0},\;\;\;\pi_{p}=1/2,\;\;\;\pi_{m}=1/2-p_{0}\;.$$
- *For $N_{S}$ odd, the exact error probability is as follows:*
(6) Pb(e)=∑k=0M12erfcNsEbM2N0(M−2k)MkπmkπpM−k
  *and can be approximated by the following:*
(7) $$P_{b}\left(e\right)≃\frac{1}{2}erfc\sqrt{\frac{p_{0}^{2}N_{s}\left(E_{b}/N_{0}\right)}{1+2\left(1-p_{0}^{2}\right)\;N_{s}\left(E_{c}/N_{0}\right)}}.$$
  *where we have the following:*
Ec=Eb/M,p0=NS−1(NS−1)/22−(NS−1)
$$\pi_{p}=\left(1+p_{0}\right)/2,\;\;\;\pi_{m}=\left(1-p_{0}\right)/2\;.$$

The proof of Theorem 1 is available in Appendix A.

In the above equations, $E_{b}/N_{0}$ is the signal-to-noise ratio for the received signal at the observed satellite. The $N_{S}$ satellites might be at different distances from the ground station and, thus, have different path losses, but the signal-to-noise ratio and the effects of interfering components in the received CDM signal for satellite *n* do not depend on the positions of the other satellites, as we assume that the HPA output power is evenly distributed among all the served satellites.

**Figure 12:**
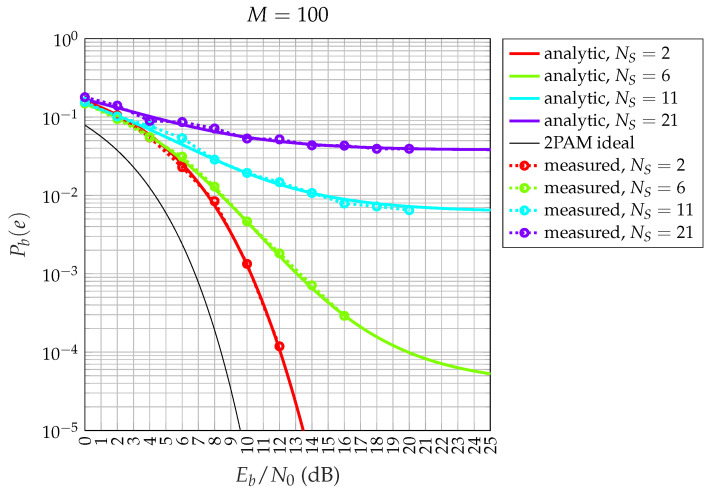
Comparison between exact theoretical (lines) and simulated (circles) CDM bit error probabilities for a saturated HPA and random spreading codes with spreading factor M=100, for $N_{S}=2,6,11,21$ satellites.

This theorem is highly significant because it enables the performance calculation analytically, as a function of the number of satellites, eliminating the need for simulations. First, we numerically validate the exact probability results of Theorem 1. In Figure 12, we compare the analytical curves obtained using Theorem 1, Equations (3), (4), and (6), with those generated through simulation. The results show perfect agreement for various numbers of satellites served. This confirms that the theoretical analysis is correct.

**Figure 13:**
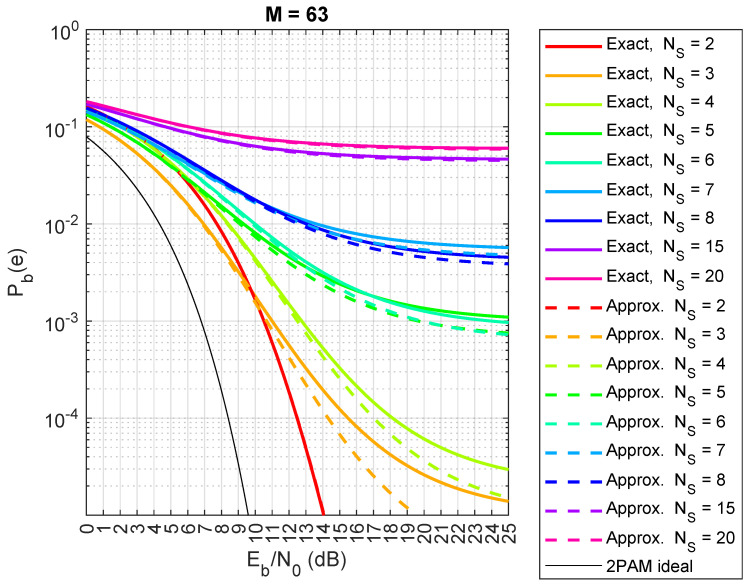
Comparison between exact theoretical (lines) and approximate (dashed lines) CDM bit error probabilities for a saturated HPA with random spreading codes, with spreading factor M=63, for $N_{S}=2,3,4,5,6,7,8,15,20$ satellites.

**Figure 14:**
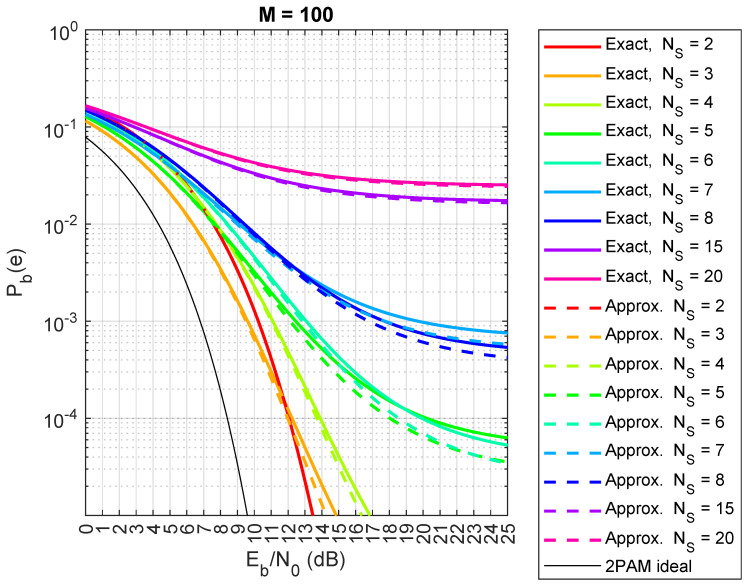
Comparison between exact theoretical (lines) and approximate (dashed lines) CDM bit error probabilities for a saturated HPA with random spreading codes, with spreading factor M=100, for $N_{S}=2,3,4,5,6,7,8,15,20$ satellites.

**Figure 15:**
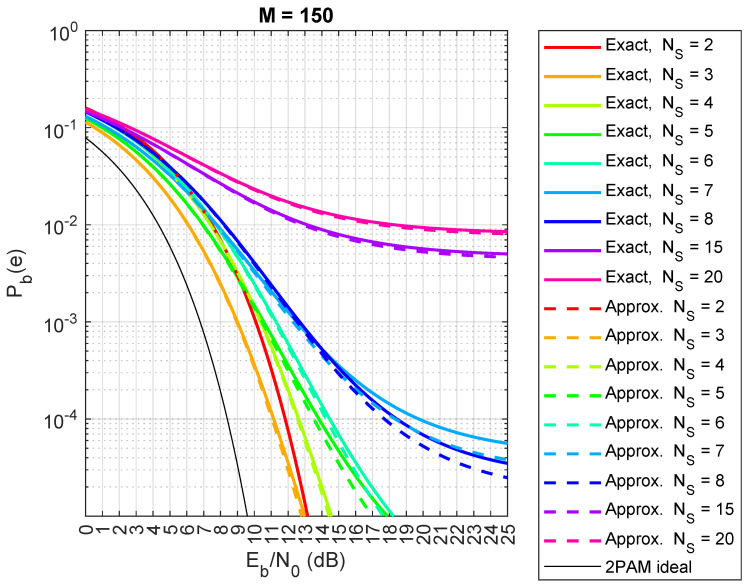
Comparison between exact theoretical (lines) and approximate (dashed lines) CDM bit error probabilities for a saturated HPA with random spreading codes, with spreading factor M=150, for $N_{S}=2,3,4,5,6,7,8,15,20$ satellites.

Now, we compare the exact and approximated expressions (Equations (5) and (7)) from Theorem 1. The results are shown in Figure 13 (spreading factor M=63), Figure 14 (M=100), and Figure 15 (M=150). Although the exact expression shows perfect agreement, there are small differences in this case. When M=63, the approximated $P_{b}\left(e\right)$ in Equations (5) and (7) actually underestimates the true value of $P_{b}\left(e\right)$, as shown in Figure 13, especially when the number of satellites, $N_{S}$, is small. However, the difference between the two formulas reduces when *M* increases to 100, as shown in Figure 14. Moreover, the approximate formula is significantly simplified and provides valuable insights into certain phenomena related to the behaviors of different numbers of satellites, as we will explore in the next section. Regarding the choice of sequences in our analysis, when dealing with a large number of satellites (thousands), pseudo-random sequences are typically preferred over algebraic sequences, like Gold sequences. However, if the pseudo-random sequences exhibit good properties, the results remain consistent.

## 5. Loss with an Increasing Number of Satellites

From the operator’s perspective, the priority is to maximize the number of satellites for a given transmitted/consumed power while meeting a certain target error rate. The analysis conducted in the previous section using Theorem 1, reported in Figure 13 (M=63), Figure 14 (M=100), and Figure 15 (M=150), leads to the following observations:

- The case $N_{S}=2$ suffers from a 3 dB loss because with a probability of 0.5 the signal is zero. At low $E_{b}/N_{0}$, the error probability for $N_{S}=3$ is, therefore, smaller than that for $N_{S}=2$, even if the interference is obviously higher. At $P_{b}\left(e\right)=10^{-2}$ and M=150—$N_{S}=4$ is better than $N_{S}=2$.
- Similar phenomena occur with $N_{S}=4$ and $N_{S}=5$: $N_{S}=5$ is better than $N_{S}=4$ at higher values of $P_{b}\left(e\right)$ (e.g., $P_{b}\left(e\right)=10^{-2}$), whereas $N_{S}=5$ is worse than $N_{S}=4$ at smaller values of $P_{b}\left(e\right)$ (e.g., $P_{b}\left(e\right)=10^{-4}$).
- The error floor for $N_{S}=8$ is smaller than the error floor for $N_{S}=7$; using the approximation for $P_{b}\left(e\right)$, we see that when $N_{S}=8$, there is a term ($1-p_{0}-p_{0}^{2}$) at the denominator, whereas the term at the denominator is $1-p_{0}^{2}$ for $N_{S}=7$; note that the two values of $p_{0}$ are not equal for $N_{S}=7$ and 8, but this is the only difference in the formula.

These considerations suggest that it would be useful to have an analytical result to calculate the loss as a function of the number of satellites. This is the result of Theorem 2 presented in this section.

Given the value ${\left(\frac{E_{b}}{N_{o}}\right)}^{*}$ [dB] necessary to achieve an error rate $P_{b}{\left(e\right)}^{*}$ for the uncoded 2-PAM, for a single satellite, the value ${\left(\frac{E_{b}}{N_{o}}\right)}^{\prime }={\left(\frac{E_{b}}{N_{o}}\right)}^{*}+Loss$ [dB] necessary to achieve the same error rate in $N_{S}$ satellites and the spreading factor *M* is given by the following results:

**Theorem** **2.**  For an uplink CDM system serving $N_{S}$ satellites with a saturated amplifier, the approximate loss in dB required to achieve a given $P_{b}{\left(e\right)}^{*}$ in the case of a spreading factor M is given by the following:

- *$N_{S}$ odd:*
$$Loss_{dB}=-10\log_{10}N_{S}-10\log_{10}\left(p_{0}^{2}-\frac{2(1-p_{0}^{2})}{M}{\left(\frac{E_{b}}{N_{o}}\right)}^{*}\right)$$
  *where we have the following:*
p0=NS−1(NS−1)/22−(NS−1).
- *$N_{S}$ even:*
$$Loss_{dB}=-10\log_{10}N_{s}-10\log_{10}\left(p_{0}^{2}-\frac{2(1-p_{0}-p_{0}^{2})}{M}{\left(\frac{E_{b}}{N_{o}}\right)}^{*}\right)$$
  *where we have the following:*
p0=NS−1NS/2−12−(NS−1).

**Figure 16:**
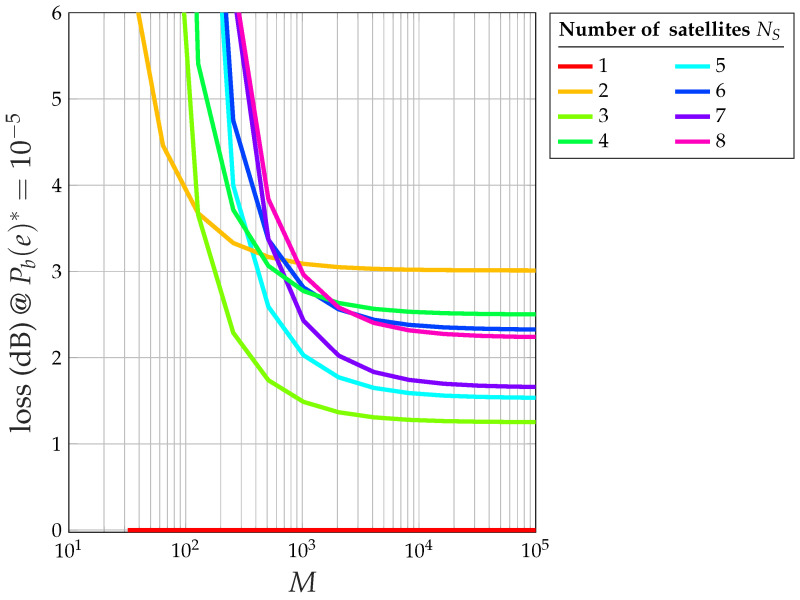
Overall loss (dB) due to interference and saturated HPA versus *M* at $P_{b}{\left(e\right)}^{*}=10^{-5}$ of the system with saturated HPA with respect to ideal 2-PAM for $N_{S}=1,\ldots ,8$.

**Figure 17:**
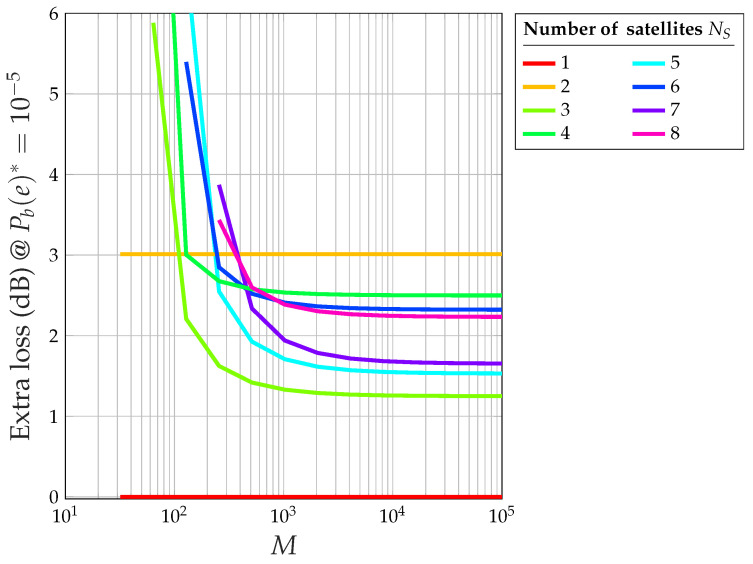
Extra loss (dB) versus *M* at $P_{b}{\left(e\right)}^{*}=10^{-5}$ exclusively due to the saturated HPA for $N_{S}=1,\ldots ,8$.

**Figure 18:**
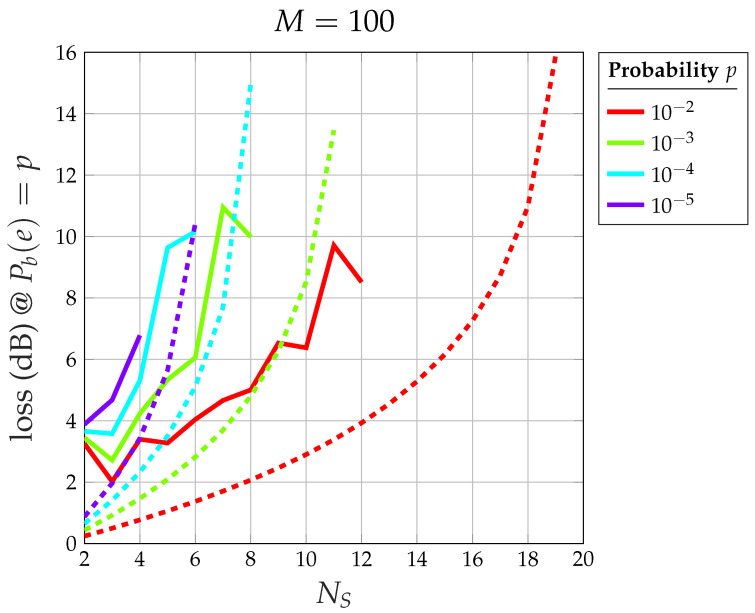
Overall loss (dB) versus $N_{S}$ at certain values of $P_{b}{\left(e\right)}^{*}$ with respect to ideal 2-PAM for M=100: solid lines refer to the system with saturated HPA, dashed lines refer to the ideal linear system.

**Figure 19:**
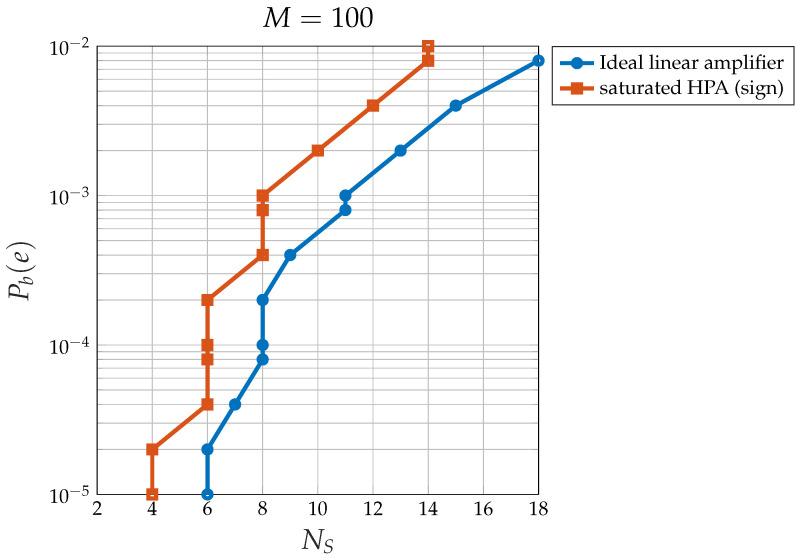
Relationship between the maximum number of satellites $N_{S}$ at $P_{b}{\left(e\right)}^{*}$ for the CDM system with saturated HPA and the linear system.

The proof of this theorem is available in Appendix B. To fully understand the impact of the HPA, it is useful to compare it with an ideal, fully linear system. Theorem 2 allows us to obtain the following results:

- Figure 16 shows the overall loss with respect to the ideal 2-PAM of the CDM system with saturated HPA at ${\left(E_{b}/N_{0}\right)}^{*}=\eta =9.58$ dB (i.e., at $P_{b}{\left(e\right)}^{*}=10^{-5}$): note that, for M→∞, an odd number of satellites gives rise to a smaller loss (less than 2 dB), whereas an even number gives a higher loss (more than 2 dB).
- Figure 17 shows the extra loss (with respect to the same CDM system and an ideal perfectly linear amplifier) due to the saturated HPA at ${\left(E_{b}/N_{0}\right)}^{*}=\eta =9.58$ dB (i.e., at $P_{b}{\left(e\right)}^{*}=10^{-5}$).
- Figure 18 shows the relationship between the number of satellites $N_{S}$ and the corresponding loss with respect to an ideal 2-PAM system at certain values of $P_{b}{\left(e\right)}^{*}$. Having set, for example, $P_{b}{\left(e\right)}^{*}=0.01$, the maximum number of satellites is 12 for the system with saturated HPA (for $N_{I}=12$, the error floor for $E_{b}/N_{0}\to \infty$ is higher than $P_{b}{\left(e\right)}^{*}=0.01$), whereas the linear system allows for 18 satellites. The relationship between $N_{S}$ and the loss for the case of saturated HPA is not as smooth as that for the case of a linear system due to the fact that two different loss equations exist for even or odd values of $N_{S}$.
- Figure 19 shows the maximum number of satellites that the system can support at the desired value of $P_{b}{\left(e\right)}^{*}$ at the satellite with the smallest signal-to-noise ratio. When the channel is nonlinear, the maximum number of satellites is understandably smaller than when the channel is linear, but the difference amounts to 2–3 satellites only.

### Discussion

The final conclusions are as follows:

- The extra loss due to the saturated HPA at $P_{b}\left(e\right)=10^{-5}$ is less than 3 dB for M>60 and $N_{S}>2$.
- For $N_{S}\to \infty$ and M→∞, the extra loss tends to 2 dB ($10\log_{10}\pi /2$).
- Overall, the loss to be factored into the link budget (to account for the presence of a nonlinear amplifier) can be safely set at 3 dB, provided that M>60, which is almost always the case.
- If a 3 dB loss is acceptable, it might be more convenient to directly use the high-power amplifier in the saturated region (or to transmit the sign of the CDM signal) for easier management of the transmitter (as explained before, since the number of served satellites changes rapidly, maintaining the amplifier in the linear zone with appropriate back-off is complex).

## 6. Conclusions

In this paper, we examined the uplink connection from a ground station to an LEO satellite constellation using code division multiplexing (CDM). Due to the rapidly changing number of visible satellites, summing CDM signals introduces challenges in maintaining an appropriate back-off without significantly compromising output power. We showed that the best performance is achieved when the power amplifier operates in saturation (where we have maximum efficiency, too). In this case, we can directly transmit the sign of the sum of the CDM signals sent to the individual satellites. We then provided an analytical calculation of system performance. Simplified expressions were also derived, and both the exact and approximate error probability expressions were validated through simulations. Additionally, we presented an analytical method for calculating system loss as a function of the number of satellites. A number of results were presented and discussed. This study offers valuable insights for assessing the performance of a ground station operating with CDM in saturation mode when serving LEO constellations, eliminating the need for extensive simulations. Additionally, it offers useful guidelines and quantitative understanding for operating the ground station when using CDM for the uplink. Future investigations could focus on the impact of the proposed approach on the initial acquisition process and the phase error at the receiver. The acquisition stage is critical for synchronizing the receiver with the transmitted signal, and further research could provide valuable insights into how the sign approach and the non-linear amplifier affect the accuracy and speed of this process. Additionally, analyzing the phase error at the receiver would be important to understand its influence on overall system performance.

## Data Availability

The data presented in this study are available on request from the corresponding author.

## Appendix A. Proof of Theorem 1

**Proof** **of** **Theorem** **1.** We assume that the receiver correctly acquired the received signal frequency and phase and that the phase jitter is small. This hypothesis allows for a simplification of the analysis, where only the in-phase component of the 2-PSK received signal is relevant.

$$s_{T}^{\prime }\left(t\right)=\sqrt{P_{max}}\mathrm{sign}\left[\sum_{i=0}^{N_{S}-1}\sum_{n}b_{i}\left(n\right)P_{T_{b}}\left(t-nT_{b}\right)\sum_{m}c_{i}\left(m\right)P_{T_{c}}\left(t-mT_{c}\right)\right]$$

The received signal is $r\left(t\right)=s_{T}\left(t\right)+n\left(t\right)$ where n(t) is white Gaussian noise with a power spectral density of $N_{0}/2$. During the *m*-th chip interval in the first-bit period, the receiver of satellite *i* evaluates the following:

$$r\left(m\right)=\frac{1}{\sqrt{T_{c}}}\int_{mT_{c}}^{\left(m+1\right)T_{c}}r\left(t\right)dt=\sqrt{P_{max}T_{c}}\mathrm{sign}\left[\sum_{j=0}^{N_{S}-1}b_{j}\left(0\right)c_{j}\left(m\right)\right]+n\left(m\right),$$

where $n\left(m\right)∼N\left(0,N_{0}/2\right)$, and then we have the following:

$$r=\sum_{m=0}^{M-1}r\left(m\right)c_{i}\left(m\right)=\sqrt{P_{max}T_{c}}\left(\sum_{m=0}^{M-1}c_{i}\left(m\right)\mathrm{sign}\left[\sum_{j=0}^{N_{S}-1}b_{j}\left(0\right)c_{j}\left(m\right)\right]\right)+n=\sqrt{P_{max}T_{c}}z+n,$$

where $n∼N(0,MN_{0}/2)$. Term *z* can be written as follows:

$$z=\sum_{m=0}^{M-1}c_{i}\left(m\right)\mathrm{sign}\left[b_{i}\left(0\right)c_{i}\left(m\right)+\sum_{j=0,j\neq i}^{N_{S}-1}d_{j}\left(m\right)\right]$$

where $d_{j}\left(m\right)=b_{j}\left(0\right)c_{j}\left(m\right)\in \left\{\pm 1\right\}$ can be assumed statistically independent of $c_{i}\left(m\right)$ for i≠j. Note that *z* is the sum of terms z(m) with three values: $c_{i}\left(m\right)$ (if the argument of the sign function is strictly positive), $-c_{i}\left(m\right)$ (if the argument is strictly negative), and 0 (if the argument is zero); therefore, *z* can take integer values from −M to *M*. Without loss of generality, we consider the transmitted symbol $b_{i}\left(0\right)=1$. Then, the error probability can be written as follows:

$$\begin{array}{cc} P_{b}\left(e\right) & =P\left(\left.r<0\right|b_{i}\left(0\right)=1\right)=P\left(\left.\sqrt{P_{max}T_{c}}z+n<0\right|b_{i}\left(0\right)=1\right)\\ \; & =P\left(\left.n>\sqrt{P_{max}T_{c}}z\right|b_{i}\left(0\right)=1\right)\\ \; & =\sum_{\zeta =-M}^{M}\frac{1}{2}\mathrm{erfc}\left(\sqrt{\frac{P_{max}T_{c}}{MN_{0}}}\;\zeta \right)P\left(z=\zeta \right)\;. \end{array}$$

Having assumed $b_{i}\left(0\right)=1$, z(m) is

$$z\left(m\right)=c_{i}\left(m\right)\mathrm{sign}\left(c_{i}\left(m\right)+\sum_{j=0,j\neq i}^{N_{S}-1}d_{j}\left(m\right)\right)=c_{i}\left(m\right)\mathrm{sign}\left(c_{i}\left(m\right)+d\right).$$

It is necessary to distinguish two cases: $N_{S}$ even and odd.

- $N_{S}$ even larger than 2.
  The random variable *d* is the sum of an odd number of terms ±1 and can never be equal to zero; instead, it is positive (i.e., ≥1) with a probability of 1/2, and negative (i.e., ≤−1) with a probability of 1/2. Therefore, we have the following:
  - z(m)=0 with a probability of the following:
    p0=P(z(m)=0)=P(d=−ci(m))=NS−1NS/2−12−(NS−1).
  - $z\left(m\right)=c_{i}\left(m\right)$ with a probability of the following:
    $$P\left(z\left(m\right)=c_{i}\left(m\right)|c_{i}\left(m\right)=1\right)=P\left(d\geq 1\right)=\frac{1}{2}$$
    $$P\left(z\left(m\right)=c_{i}\left(m\right)|c_{i}\left(m\right)=-1\right)=P\left(d\geq 3\right)=\frac{1}{2}-p_{0}\;.$$
  - $z\left(m\right)=-c_{i}$ with a probability of the following:
    $$P\left(z\left(m\right)=-c_{i}\left(m\right)|c_{i}\left(m\right)=-1\right)=P\left(d\leq -1\right)=\frac{1}{2}$$
    $$P\left(z\left(m\right)=-c_{i}\left(m\right)|c_{i}\left(m\right)=1\right)=P\left(d\leq -3\right)=\frac{1}{2}-p_{0}\;.$$
  Overall, we have the following:
  - z(m)=0 with a probability of $\pi_{0}=p_{0}$.
  - z(m)=1 with a probability of $\pi_{p}=p_{p}=1/2$.
  - z(m)=−1 with a probability of $\pi_{n}=p_{n}=1/2-p_{0}$.
  If there are $k_{0}$ terms z(m) equal to 0, then *z* takes values between $-M+k_{0}$ and $M-k_{0}$; if there are $k_{1}$ terms equal to −1, then $z=\left(M-k_{0}\right)-2k_{1}$. The exact error probability for an even $N_{s}$ is as follows:
  (A1) $$\begin{array}{cc} P_{b}\left(e\right)=\sum_{k_{0}=0}^{M}\sum_{k_{1}=0}^{M-k_{0}} & \frac{1}{2}\mathrm{erfc}\left(\sqrt{\frac{P_{max}T_{c}}{MN_{0}}}\left(M-k_{0}-2k_{1}\right)\right) \end{array}$$
  (A2) $$\begin{array}{cc} \; & \times \frac{M!}{k_{0}!k_{1}!\left(M-k_{0}-k_{1}\right)!}\pi_{0}^{k_{0}}\pi_{m}^{k_{1}}\pi_{p}^{M-k_{0}-k_{1}}. \end{array}$$
  The exact expression above can be approximated using the central limit theorem on *z*: it represents the sum of *M* (typically large) i.i.d. random variables z(m) with the mean and variance as follows:
  $$\mu_{z(m)}=\pi_{0}\times 0+\pi_{p}\times 1+\pi_{n}\times \left(-1\right)=p_{0}$$
  $$E\left\{z{\left(m\right)}^{2}\right\}=1-\pi_{0}=1-p_{0};\;\;\;\sigma_{z(m)}^{2}=1-p_{0}-p_{0}^{2}.$$
  Therefore, $z∼N(Mp_{0},M\left(1-p_{0}-p_{0}^{2}\right))$ and
  $$r=\sqrt{P_{max}T_{c}}z+n∼N\left(\sqrt{P_{max}T_{c}}Mp_{0},\;M\left(1-p_{0}-p_{0}^{2}\right)P_{max}T_{c}+MN_{0}/2\right).$$
  The error probability is reduced to the following:
  (A3) $$P_{b}\left(e\right)=P\left(r<0|b_{i}\left(0\right)=1\right)≃\frac{1}{2}\mathrm{erfc}\sqrt{\frac{P_{max}T_{c}Mp_{0}^{2}}{2\left(1-p_{0}-p_{0}^{2}\right)P_{max}T_{c}+N_{0}}}$$
  Note that when $N_{S}$ is even, the average power at the output of the saturated amplifier is
  $$P_{out,av}=P_{max}\left(1-{\tilde{\pi }}_{0}\right)$$
  where
  π˜0=NSNS/212NS.
- $N_{S}=2$
  In this case, having set i=0, without loss of generality, we have the following:
  $$z\left(m\right)=c_{0}\left(m\right)\mathrm{sign}\left(c_{0}\left(m\right)+d_{1}\left(m\right)\right).$$
  The term z(m)=0 if $d_{1}\left(m\right)\neq c_{0}\left(m\right)$, which occurs with a probability of
  $$p_{0}=1/2.$$
  If $d_{1}\left(m\right)=c_{0}\left(m\right)$, which occurs with a probability of $\pi_{p}=1/2$, then z(m)=1. Therefore, *z* is the sum of *M* terms that can take values 1 or 0 with the same probability. The exact error probability is as follows:
  Pb(e)=∑k=0M12erfckPmaxTcMN0Mk12M
  The random variable z(m) has a mean value, mean square value, and variance
  $$\mu_{z(m)}=p_{0}=\frac{1}{2},\;\;\;E\left\{z^{2}\left(m\right)\right\}=1-p_{0}=\frac{1}{2},\;\;\;\sigma_{z(m)}^{2}=1-p_{0}-p_{0}^{2}=\frac{1}{4},$$
  respectively. The approximate error probability given in (A3) applies also to the case $N_{S}=2$.
- $N_{S}$ odd
  The random variable *d* is the sum of an even number of terms ±1 and is zero with a probability of the following:
  p0=P(d=0)=NS−1(NS−1)/22−(NS−1),
  it is positive (i.e., ≥2) with a probability of the following:
  $$p_{p}=P\left(d\geq 2\right)=\frac{1-p_{0}}{2},$$
  it is negative (i.e., ≤2) with a probability of the following:
  $$p_{n}=P\left(d\leq -2\right)=\frac{1-p_{0}}{2}.$$
  Therefore
  - z(m)=1 with a probability of $p_{0}$.
  - $z\left(m\right)=c_{i}\left(m\right)$ with a probability of $p_{p}$.
  - $z\left(m\right)=-c_{i}$ with a probability of $p_{n}=p_{p}$.
  Overall, *z* is the sum of *M* random variables z(m) such that we have the following:
  - z(m)=1 with a probability of $\pi_{p}=p_{0}+p_{p}=\left(1+p_{0}\right)/2$.
  - z(m)=−1 with a probability of $\pi_{m}=p_{p}=\left(1-p_{0}\right)/2$.
  Therefore, the error probability for $N_{s}$ odd is as follows:
  (A4) $$\begin{array}{cc} P_{b}\left(e\right) & =\sum_{k=0}^{M}\frac{1}{2}\mathrm{erfc}\left(\sqrt{\frac{P_{max}T_{c}}{MN_{0}}}\left(M-2k\right)\right)\times \frac{M!}{k!(M-k)!}\;\pi_{m}^{k}\;\pi_{p}^{M-k}. \end{array}$$
  Note the following:
  $$\mu_{z(m)}=\pi_{p}\times 1+\pi_{n}\times \left(-1\right)=p_{0}$$
  $$E\left\{z{\left(m\right)}^{2}\right\}=1;\sigma_{z(m)}^{2}=1-p_{0}^{2},$$
  and $z∼N(Mp_{0},M\left(1-p_{0}^{2}\right))$ and
  $$r=\sqrt{P_{max}T_{c}}z+n∼N\left(\sqrt{P_{max}T_{c}}Mp_{0},\;M\left(1-p_{0}^{2}\right)P_{max}T_{c}+MN_{0}/2\right).$$
  The error probability is reduced to the following:
  (A5) $$P_{b}\left(e\right)=P\left(r<0|b_{i}\left(0\right)=1\right)≃\frac{1}{2}\mathrm{erfc}\sqrt{\frac{P_{max}T_{c}Mp_{0}^{2}}{2\left(1-p_{0}^{2}\right)P_{max}T_{c}+N_{0}}}.$$
  Note that when $N_{S}$ is odd, the average power at the output of the saturated amplifier is equal to the maximum power:
  $$P_{out,av}=P_{max}.$$

We now introduce $E_{b}$ (energy per bit) in (A2)–(A5). Note that during a time interval $T_{b}=MT_{c}$, the number of transmitted bits, $N_{S}$, corresponds to the number of satellites to be served. Two cases should be considered:

A. The amplifier is nonlinear and at saturation, in which case, $E_{b}/N_{0}$ is set according to the maximum power: $N_{S}E_{b}=P_{max}MT_{c}$.
B. The amplifier is ideal and linear, in which case, $E_{b}/N_{0}$ is set according to the average power: $N_{s}E_{b}=P_{av}MT_{c}=P_{max}MT_{c}\left(1-{\tilde{\pi }}_{0}\right)$. Recall that ${\tilde{\pi }}_{0}=0$ for $N_{s}$ odd.

The difference in the definition of the signal-to-noise ratio in the two cases is
  $$10\log_{10}\left(1-{\tilde{\pi }}_{0}\right),$$
  which is negligible when $N_{S}$ is large, but is equal to 3 dB when $N_{S}=2$.

Since, in this application, we want the worst-case error probability in the presence of a nonlinear amplifier, we must consider **case A**. By substituting $P_{max}T_{c}$ with $N_{s}E_{b}/M$ and using $E_{c}=E_{b}/M$ in Equations (A3) and (A5), Equations (5) and (7) are obtained. □

## Appendix B. Proof of Theorem 2

**Proof** **of** **Theorem** **2.** We use the approximated bit error probabilities in Equation (5) and (7). For $N_{s}$ even, assuming that the CDM system with the saturated HPA fed by the sign of the CDM signal has the same error probability as the ideal 2-PAM system, then

$$\frac{1}{2}\mathrm{erfc}\sqrt{\frac{p_{0}^{2}N_{s}{\left(\frac{E_{b}}{N_{o}}\right)}^{*}Loss}{1+2\left(1-p_{0}-p_{0}^{2}\right)/M\;N_{s}{\left(\frac{E_{b}}{N_{o}}\right)}^{*}Loss}}=\frac{1}{2}\mathrm{erfc}\sqrt{{\left(\frac{E_{b}}{N_{o}}\right)}^{*}}$$

$$p_{0}^{2}N_{s}{\left(\frac{E_{b}}{N_{o}}\right)}^{*}Loss={\left(\frac{E_{b}}{N_{o}}\right)}^{*}\left[1+\frac{2(1-p_{0}-p_{0}^{2})}{M}\;N_{s}{\left(\frac{E_{b}}{N_{o}}\right)}^{*}Loss\right]$$

$$N_{s}Loss=\frac{1}{p_{0}^{2}-\frac{2(1-p_{0}-p_{0}^{2})}{M}\;{\left(\frac{E_{b}}{N_{o}}\right)}^{*}}.$$

In a similar way, the loss for $N_{S}$ odd is obtained. The loss value found is an approximation of the exact loss since (5) and (7) are approximations of the exact error probability. □
